# Statistical consideration when adding new arms to ongoing clinical trials: the potentials and the caveats

**DOI:** 10.1186/s13063-021-05150-7

**Published:** 2021-03-10

**Authors:** Kim May Lee, Louise C. Brown, Thomas Jaki, Nigel Stallard, James Wason

**Affiliations:** 1 MRC Biostatistics Unit, School of Clinical Medicine, University of Cambridge, Cambridge, CB2 0SR UK; 2 Pragmatic Clinical Trials Unit, Queen Mary University of London, Yvonne Carter Building, 58 Turner Street, London, E1 2AB UK; 3 MRC Clinical Trials Unit, University College London, 90 High Holborn 2nd Floor, London, WC1V 6LJ UK; 4 Medical and Pharmaceutical Statistics Research Unit, Department of Mathematics and Statistics, Lancaster University, Lancaster, UK; 5 Statistics and Epidemiology, Division of Health Sciences, Warwick Medical School, University of Warwick, Coventry, CV4 7AL UK; 6 Population Health Sciences Institute, Baddiley-Clark Building, Newcastle University, Richardson Road, Newcastle upon Tyne, UK

**Keywords:** Adding arms, Bias, Error rates, Multiplicity, Platform trials

## Abstract

**Background:**

Platform trials improve the efficiency of the drug development process through flexible features such as adding and dropping arms as evidence emerges. The benefits and practical challenges of implementing novel trial designs have been discussed widely in the literature, yet less consideration has been given to the statistical implications of adding arms.

**Main:**

We explain different statistical considerations that arise from allowing new research interventions to be added in for ongoing studies. We present recent methodology development on addressing these issues and illustrate design and analysis approaches that might be enhanced to provide robust inference from platform trials. We also discuss the implication of changing the control arm, how patient eligibility for different arms may complicate the trial design and analysis, and how operational bias may arise when revealing some results of the trials. Lastly, we comment on the appropriateness and the application of platform trials in phase II and phase III settings, as well as publicly versus industry-funded trials.

**Conclusion:**

Platform trials provide great opportunities for improving the efficiency of evaluating interventions. Although several statistical issues are present, there are a range of methods available that allow robust and efficient design and analysis of these trials.

## Background

Platform trial designs offer an innovative approach to increase the efficiency of the drug development process with great potential to positively change the conduct of clinical trials. This approach allows adding and dropping research arms throughout the course of an interventional study via protocol amendments. Esserman et al. described that “Amendments to already-approved protocols are faster and more efficient, avoiding the need for repeated review of all study procedures, creating a seamless process that avoids disruption of enrolment as drugs enter and leave the trial [1].” This shows that the overall time and the cost spent on evaluating new interventions might be reduced when there is a relevant platform trial. From the perspective of patients, participating in a platform trial may lead to a higher chance of receiving an experimental treatment which may be appealing and lead to higher recruitment.

The benefits of a platform approach are most prevailing for disease areas where (i) there are multiple candidate treatments and new ones being developed, (ii) the recruitment rate can support a platform trial, and (iii) an informative endpoint is observed relatively quickly that can be used to make adaptations (for adaptive platform trials). The features and advantages of platform trials have been recently illustrated by trials for COVID-19 [2, 3]. Trial examples that have considered a platform approach include RECOVERY [4] that evaluates a range of potential treatments for hospitalized patients with suspected or confirmed COVID-19, and PRINCIPLE [5] that evaluates treatments for older people with symptoms of possible COVID-19.

Nevertheless, allowing adding of new research comparisons increases the operational burdens and complexities of trial conduct [6–11]. The challenges in developing and implementing novel clinical trial designs have also been discussed in a wider context [12–15]. In the statistical literature, methodological aspects of dropping arms have been well explored [16–25]. The issues arising from adding new research comparisons remain less considered. To the best of our knowledge, Cohen et al. [26] is the only review focused on adding arms. They identified seven publications that discussed methodological considerations when adding arms to ongoing trials, and eight confirmatory two-arm trials that have added a treatment arm (most were not in the initial plan of the trials). From the practical perspective, Schiavone et al. [6] have presented some (non-statistical) criteria for decision-making when adding new arms.

Here, we focus on phase II and phase III trials that compare the benefits between the research interventions and a control arm (with either placebo, active control, or standard of care as the treatment) for one patient population and a single disease. We do not specifically consider trials that involve multiple subgroups such as basket trials, umbrella trials, and adaptive enrichment trials, though we expect most of the arguments would be similar when adding new arms to these types of studies. For brevity, we define “new research comparisons” as the inference about the comparisons between the newly added interventions and the control treatment. By treatment effect, we refer to the difference between the treatment effect of one research intervention and the treatment of the control arm. We do not consider the implications of comparisons between different research interventions.

With the increased recent use of platform trial designs and additional methodology work considering statistical issues, it is timely to review the impact on statistical inference of adding arms. In this paper, we discuss some additional issues, such as changing the treatment of the control arm and how patient eligibility would complicate the trial design and analysis, which have not been previously covered by Cohen et al. [26]; we also summarize some recent relevant work. In addition, we cover recent insights from the more generic statistical literature that pave the way for future methods for platform trials. Lastly, we remark how statistical considerations may vary when using the platform trial approach for phase II and phase III trial settings, as well as from the perspective of publicly and industry-funded studies.

### Background of trial settings

We consider a randomized trial that initially explores the inference about at least one research comparison relative to a common control group. After the study has commenced but before the end of recruitment, a new intervention is added allowing a new research comparison following this amendment. We refer to stage 1 and stage 2 as before and after the new arm is added to the study, respectively. Each research comparison has an associated null hypothesis representing no true treatment effect.

## Issues in the use of platform trials

### The presence of time trends

One of the potential concerns when implementing platform trials is that the effect of a treatment (either an intervention or the treatment of the control arm) may vary with time, since their lifetime is often longer than fixed trials. This happens for example when there is a learning curve amongst the study personnel or when usual care in general practice changes with time. Some authors [27] described this change as a chronological bias, and others describe this as a time trend. It causes issues in making inference when the estimate of each mean is not consistent in the sense that the bias might not be offset when computing the mean difference. We note that in fixed trials that have long durations, this is also a concern, unless the assumption that arms are affected equally holds. It could be more of an issue when an arm is added and the analysis approach naively compared all data on the control arm with the new arm. We discuss the impact of such a trend on the inference about the new research comparison in the “Analysis approaches” section.

### Impact of adding arms on the initial research comparisons

Another potential problem of implementing platform trials arises from the fact that the initial research comparison does not account for the fact that new interventions have been added to the trial. More specifically, a change in the trial design may lead to different treatment effects in stages 1 and 2 if stage 1 and stage 2 patients respond differently to a treatment (either one of the initial interventions or the control treatment). This may be due to the fact that a different “type” of patients participate in stage 2, e.g. these patients were not happy with the initial treatment options but are willing to participate now that a new option is available. Consequently, the estimates of treatment effects and of the variances of the estimates may be affected, leading to a spurious result for the investigation.

## Inference about the initial research comparisons

Valid inference is of major concern to regulatory authorities, and hence, buy-in by regulatory authorities to the inferential approach taken at the outset of a platform design is paramount. In this section, we focus on the inference about the initial research comparisons. We delegate the discussion on the inference about the new research comparisons to the next section.

There have been methods proposed to account for the variability of treatment effect being affected by adding new arms. Elm et al. [28] find that a linear model adjusting for a stage effect outperforms a simple *t* test and an adaptive combination test for trials with a normal endpoint. Potentially, the varying variability in the responses across different stages might be resolved by using a robust variance estimator in the test statistics. The work by Rosenblum and van der Laan [29] indicates that for an unbiased estimate of treatment effect, using a sandwich estimator to estimate the variance of treatment effect when the analysis model is misspecified could preserve the type I error rate at the nominal value asymptotically. However, they show by simulation that this approach leads to a smaller power compared to using the true population model. Other approaches worth considering have been proposed by Chow et al. [30] and Yang et al. [31], who study the inference when the target population deviates following a protocol amendment. Specifically, Chow et al. [30] explore measures that reflect the differences between the actual population and the original target population whereas Yang et al. [31] focus on the binary outcomes and propose estimates that link the response rates of populations following protocol amendment.

Alternatively, one may consider a randomization-based test (see, e.g. Cox and Reid [32], section 2.2.5), especially when the properties of the analytical estimates (of the treatment effect and its variance) are of concern. The notion of randomization-based inference is that under the null hypothesis, i.e. when there is no true treatment effect, the observed difference between the treatment and the control group is due to the random allocation. Specifically, the null hypothesis states that the distribution of the responses of one group is the same as that of another group. Simulation is used to construct a reference distribution of a test statistic under the null scenario. Given an observed test statistic, i.e. computed using the observed data, this reference distribution is used for testing the null hypothesis in a way similar to the standard *t* test. We note that for testing the initial research comparison using this approach, care is needed when generating the reference distribution since it also requires the assumption that responses are independent and identically distributed. In particular, the reference distribution needs to reflect the random allocation sequences of stage one and of stage two for the initial arms, which implicitly would account for the presence of the newly added arm. This approach may not be favoured over other approaches based on a parametric model since the latter would have higher power when their assumptions are met. However, it is unclear which approach is better in the context of platform trials when the responses of the same arm across the two stages could come from different distributions.

## Inference about the new research comparison

Additional research comparisons have a profound impact on the characteristics of a platform study, and consequently, careful considerations, in partnership with regulatory agencies, should be given to aspects such as analysis and error rate control.

### Analysis approaches

We now discuss the inference about the new research comparison. Recall that the control arm has responses in both stages 1 and 2, whereas the new arm has responses only in stage 2. Options for the analysis are (1) use the control data of both stages and (2) use only the control data of stage 2. In the ideal situation, i.e. there is no time trend and the distributions of stage 1 and stage 2 responses are known and identical, option 1 would increase the precision of the estimate due to the smaller estimated variance for the estimated effect of the control treatment. Assuming known variance parameter for the normal outcome, Lee and Wason [33] show that given a treatment effect, the gain in the marginal power of option 1 depends on the timing of adding arm: the increase in the marginal power is relatively larger when the arm was added at an earlier time point than at a later time point. However, when there is a trend in the study, option 1 leads to bias in estimation that consequently causes the type I error rate and the marginal power to deviate from the corresponding nominal values, whilst the root mean squared error of the estimated treatment effect is smaller than that from option 2 when the time trend is not too large. Many might think that option 1 can increase the marginal power of the hypothesis test, but some researchers [33–35] have highlighted that the gain in the power would not be possible with a strict control of type I error rate when the rejection boundary of the standard two-arm trial is used. This indicates that the benefit of option 1 is more appealing when aiming to use the trial data for generating exploratory evidence about the efficacy of treatments (e.g. through building predictive models which trade bias for a reduction in variance), but not when a strict control of error rates is required for the inference of the trial population. As discussed in the “Conclusion” section, this may present a barrier to use in registration trials.

In contrast, option 2 may yield inference that is more robust to time trends. For example, advancement in usual healthcare may affect the baseline characteristics of patients as well as how they respond to a treatment; improvement in diagnosis procedures may lead to the enrolment of patients who are more representative than those enrolled in the past. These inherent factors may cause concern about the similarity between stage 1 and stage 2 patients, though randomization could potentially minimize the impact of these uncontrollable factors if the effect over time is the same across all arms. However, as the randomization procedure used in platform trials generally changes when a new arm is added, the patients of the newly added arm may not be comparable to the patients of the control arm who were randomized in stage 1. Since stage two patients are randomized to all arms during the same period, using only the control data of stage 2 patients in the analysis about the new research comparison is likely to lead to more reliable conclusions than using option 1.

We note that option 1 is analogous to using historical control data in a two-arm randomized controlled trial [34–44], where Bayesian approaches have been explored to study the gain in utilizing the historical control data, and option 2 to using only the collected data from that trial. Moreover, option 1 might be more beneficial if some randomization procedures can maintain the balance in patient characteristics and responses across different stages. Future work is required to explore this in the spirit that is similar to Feng and Liu [45], who assume the responses of populations across different stages are associated with some known covariates in their proposal of group sequential test procedure.

### Error rate of the new comparisons

For the type I error rate of the new research comparison, the same rate as the initial comparison may be used as in the STAMPEDE trial [46]. This is legitimate when the research comparisons are treated as independent research investigations, with a type I error rate pre-specified for each hypothesis. The whole platform trial can be thought of as a multi-faceted tool that evaluates multiple interventions simultaneously and in a continuous manner whenever new interventions are ready for the evaluation. The only inconsistency with a platform trial being thought of in this way is that the data of the control group is utilized in all research comparisons that are active over the same period. This shared control group means that test statistics are positively correlated, which actually reduces the total chance of making at least one type I error compared to if the trials were run separately with distinct control groups, though the overall error rate is still larger than the individual type I error rate of each test. The drawback is that if the responses of the control group in a platform trial are such that one of the null hypotheses is rejected incorrectly, it is likely that other hypotheses would also be rejected incorrectly.

Proponents of adjusting the rejection boundary for testing multiple hypotheses often illustrate the issue with a measure that describes the total chance of making any type I error, e.g. family-wise error rate (FWER) and per-comparison error rate. When we consider platform trials as a whole, adjustment for multiplicity can be challenging since the number of research comparisons varies with time and it can be hard to envisage the frequency and the timing of adding arms. As the conventional approaches require the grouping of hypotheses for which we wish to control the FWER, which is defined as the chance of rejecting at least one null hypothesis, it might not be straightforward to extend the grouping of hypotheses to cover for the new research comparisons. Moreover, the control of error rate depends on the allocation ratio, the rules of dropping intervention arms, and whether all intervention arms finish recruitment at about the same time. Currently, there is no explicit guidance or framework on how this should be achieved in the setting of platform trials. Investigating different ways of grouping the hypotheses and their implication on the goal of the trial (or power) and with different procedures such as *p* value combination approaches [47–50] and closed-testing procedures [51] are an area for future research.

Wason et al. [52] have explored the impact of adding new arms on the FWER in a two-stage setting using a design that allows for early stopping. Without adjusting the rejection boundaries of the testing procedure, they find that adding new arms causes an inflation of the FWER over the nominal value. For trials that do not allow for early stopping, Choodari-Oskooei et al. [53] show that the standard Dunnett’s test can be extended to control the FWER when a new arm is added in for stage 2. The idea is to adjust the correlated test statistics by a factor that reflects the size of the shared control group that are used in all research comparisons. This is analogous to considering a multi-arm design with some of the intervention arms are delayed for recruitment. Bennett and Mander [54] explore the control of the FWER comprehensively. They consider maintaining the same marginal power for each research comparison and adjusting the rejection boundary in light of having a larger sample size per arm when a new intervention is added. They also propose algorithms to compute the allocation ratio when all arms finish recruiting at the same time point and at different time points respectively. These recent works focus only on the initial design of platform trials when new interventions are added. They do not explore the feature of dropping arms within the platform designs of more than two stages. Burnett et al. [55] on the other hand use a conditional error approach in the spirit of Magirr et al. [56] to achieve FWER control when adding arms to a platform trial that also allows dropping of arms. Such an approach can lead to conservative inference when many arms are added to an ongoing trial.

We remind readers that when the number of hypotheses is large, some approaches (e.g. Bonferroni correction) may lead to strict rejection thresholds and unacceptably low power. The control of false discovery rate (FDR), which is defined as the proportion of rejected null hypotheses that are false, might be more appropriate for situations where the number of hypotheses is large. Examples of multiple testing methods that control FDR include the Benjamini and Hochberg procedure [57], the Benjamini and Yekutieli procedure [58], and the adaptive Benjamini and Hochberg procedure [59]. Most of the current approaches estimate and control the FDR at the design stage, assuming all test statistics are available at the end of the trial. This may not be appropriate for platform trials where new research comparisons are added in a sequential manner. Nevertheless, some researchers have proposed approaches that aim to resolve this limitation in recent years by considering a scenario where each hypothesis is tested in a sequential manner and without the knowledge of other hypotheses that would arise in another period of time [60, 61]. The solution is based on the idea of using a budget function that describes the error rate. Specifically, the budget [62] is spent when a hypothesis is not rejected, and a return is added to the budget when a hypothesis is rejected. Robertson and Wason [63] have compared several of these approaches by simulation studies, with a platform trial as one of the illustrations.

Regulatory agencies have taken different views regarding the question of controlling FWER and pairwise error rates following broadly the reasoning outlined in Woodcock and LaVange [64]. At the same time controlling for FDR was not broadly accepted by regulators at the time of writing.

## Practical considerations

### Changing the control arm

We now discuss the possibility of a change of treatment in the control arm of a platform trial. In addition to gradual changes over time, replacing the treatment in the control arm with another treatment could cause a step change. For instance, when an intervention is found to be definitively more effective than the current treatment of the control arm, there would be ethical concerns in light of not replacing the control treatment for the future patients in the trial. However, if the control treatment is replaced, that may make redundant the patients who were recruited before the transition, even if the trial was suspended whilst the transition takes place.

Moreover, the research question may need to be broadened or revised if the control treatment has changed, e.g. “compare the effectiveness of treatment X to control treatment 1” is broadened to “compare the effectiveness of treatment X to the treatments of the control arm (either control treatment 1 or other new control treatments that emerge during the active period of treatment X)”. A stratified analysis might be considered here, where the data of an intervention and the control arms is stratified according to the time when there is a change to the design (either when the comparator is changed, or a new arm is added). In other words, all available data are used to compare the research intervention with each control respectively, which may lead to several heterogeneous estimated treatment effects for a research comparison (depending on how many changes have been made to the design and the nature of the control treatments). In this case, a hierarchical modelling approach [65] might be appropriate to provide robust inference in the sense of doing a network meta-analysis. Investigating analysis approaches as such is an area for future research. Note that if the interest lies in comparing the intervention to the new control treatment only, the discussion in the “Analysis approaches” section applies analogously, where the new control treatment can be considered as the added arm whilst the intervention arm consists of two groups: before and after the introduction of new control treatment.

### Patient inclusion and exclusion criteria

It is possible that some patients are not eligible for all interventions (due to unacceptable safety risks in some patient subgroups for example). With multi-arm designs, although it may cause difficulties in interpretation and challenges in estimating the correlation structure of the test statistics, the analysis plan can describe how the information from such patients be utilized when making an inference. For platform trials, it is not obvious how this problem might be overcome when patients are recruited continuously to the control arm: including patients with such background in a standard analysis may distort the inference (see the discussion in the “Impact of adding arms on the initial research comparisons” section); excluding them may increase the risk of having selection bias. Moreover, excluding the responses of these patients in the analysis of the initial comparison would mean recruitment of more patients who have the same trait to the patients in stage one is required. This may cause complications in managing the control arm as well as its required sample size for a particular period, since the sample size is dependent on the prevalence rate of patients with such complications. Investigating how best to utilize comparable patients in the analysis and compute the required sample size are areas for future research.

Trials that have encountered such a challenge include the RECOVERY [4], RECOVERY-RS [66], and STAMPEDE [46]. These trials use a randomization system that is capable of randomizing patients between a limited subset of treatments according to the patient background and labelling these patients for the purpose of the analysis.

### Randomization: allocation ratio

As discussed, the inference about a research comparison can be distorted when the differences in the characteristics of the comparator groups are not accounted for in the analysis. Randomization can minimize bias caused by the presence of confounding factors (i.e. unobserved variables that affect how patients respond to treatments) in advance of data analysis when the allocation ratio is preserved in terms of patient ordering. The recently proposed error rate control frameworks [53, 54] allow unequal allocation ratios when new arms are added. Yet, no one has explored how best to choose the unequal ratio in favour of the new arms under various settings or from the perspective of stakeholders.

To our knowledge, many platform trials (e.g. EVD [67], ISPY-2 [68], GMB Agile [69], and REMAP-CAP [70]) have included response adaptive randomization rules [71–73]. Some of the Bayesian response adaptive randomization rules aim to randomize more patients to the putatively superior arms based on the trend of the accrued data in a trial, but their applications to real trials have raised some controversies [74–78], some of which are partly due to the drawbacks of some algorithms and/or the risk of experiencing an unknown time trend in the trial [79]. Nevertheless, Ventz et al. [80] have compared several randomization procedures for trials that add arms in more details. Apart from discussing a balanced randomization algorithm and two data-driven randomization algorithms, Ventz et al. [80] incorporate early stopping rules into the trial designs (which maintain the type I error rate of each research comparison) and introduce a Bootstrap procedure for making inference when the latter two algorithms are implemented. The idea of employing a Bootstrap procedure is to overcome the challenges in specifying analytical distributions for the estimates when the allocation ratio is data-driven; such a procedure can produce confidence intervals of an estimate and is one of the approaches for conducting a randomization-based test that is discussed in the “Inference about the initial research comparisons” section.

Future work would be useful for evaluating the robustness of data-driven randomization approaches when there is a non-negligible time trend in platform trials in a similar way to the work of Jiang et al. [81], who explore the presence of time trend in a two-arm setting when Bayesian response adaptive randomization is employed. Comparisons with other non-adaptive randomization methods, such as minimization and block randomization, may also be made to evaluate the trade-off of various aspects, e.g. patient benefit and complexity in implementation. Minimizing the presence of other biases, such as selection bias and contamination bias (which is defined as the bias in inference due to control patients who are non-eligible for a particular intervention arm being included in the analysis of that arm), from the perspective of randomization is also an important area for future research, for the reason discussed in the “Patient inclusion and exclusion criteria” section. The ERDO framework [82] and other approaches [83] might be extended to provide guidance on selecting a randomization procedure for implementation in platform trials.

### Operational bias due to observed result of some interventions

Another challenge in platform trials could be that revealing the results of the initial interventions may risk operational bias, due to continuous recruitment of patients to the control arm. Depending on early results in the trial, the recruitment approach may change, and the way of intervention delivery or the measurement of responses may be affected. Consequently, the patients recruited before and after the result dissemination may be different, leading to the issues mentioned in the preceding discussion. It could also be the case that when the characteristics of the control treatment are revealed, some concerns about research comparisons that are active in recruitment may arise, for example, if the observed effect of the control treatment is lower than that assumed in the sample size calculation of other research comparisons. This observed effect could be due to a random chance, but the trial team may conclude that other research comparisons might be underpowered or overpowered. Subsequently, a revision of the design may lead to a change in design, e.g. revision of the sample size to match the observed characteristics of the control treatment. A pragmatic approach to avoid some of these issues might be as follows: pre-specify rules at the design stage, e.g. sample size recalculation [84–86] when new arms are added using the promising zone design [87], and exploration of different scenarios by simulation to ensure that the error rate control is within the acceptable limits of the platform trials. Future work is required to extend the methodology for sample size re-estimation in such a direction since most of the approaches are applicable to fixed trial designs in a blinded or unblind manner [88].

Despite the fact that operational bias is difficult to be minimized in practice, one may conduct sensitivity analysis to explore the robustness of the design amendments (e.g. sample size calculation or randomization approaches) and the finding of the research comparisons by simulation study. We note that the reporting guidelines [89–91] developed for randomized trials that use adaptive designs might provide useful principles that are applicable to publishing the result of the research comparisons that have finished recruitment. Examples include reporting of methods used to account for changes made in the trial in the analysis, methods to control for operational biases that might arise from results being available, and how randomization methods were updated during the trial after interim analyses.

### Trade-off between costs, benefits, and risk

We have presented what statistical adjustments are required when adding arms to ongoing trials through a platform approach. Apart from the potential risk of the negative impact on ongoing comparisons, the trade-off between costs, benefits, and operational challenges would play an important role in making the decision, even if it is established that adding arms to an ongoing trial is feasible in principle. Lee et al. [92] show that interim observation or results of the initial research comparisons might support the decision-making process. For instance, the interim observation of the initial arms may suggest that it is not worth adding in a new research arm. An extension to this framework might be to account for the disease’s prevalence as well as for different types of outcomes, with or without follow-up requirements.

The other option that may be more favourable in terms of practicality is to conduct another trial. In some cases, the simplicity in trial management (e.g. financial and staffing are predictable in trials that use fixed designs) can be more appealing than the, potentially, marginal benefit of adding new arms. Moreover, investigators have the flexibility in choosing how the new trial is being conducted and save the effort in researching and evaluating ongoing trials that seem relevant. Another reason why conducting a new trial might be favoured is that there could be a perceived hierarchy in the interventions. Taking the recent outbreak of Covid-19 as an example, whilst there are lots of drugs that potentially could be repurposed, there was consensus that two of them are most promising. So, instead of adding arms to ongoing studies, the clinical teams of some trials [93, 94] have decided to start a new study with different centres.

## Conclusion

In this paper, we have reviewed statistical issues that arise in platform clinical trials, which allow new research arms to be added whilst the trial is in process. The benefits of this approach are compelling: it allows a quicker evaluation of new interventions whilst benefiting from much of the statistical efficiency gained by multi-arm multi-stage trials. However, there are statistical complexities that cause issues with bias in the estimation, type I error, power, or interpretability of the trial.

The platform approach has clear benefits in both phase II and III settings. Many of the statistical issues we have explored in the paper will apply differently in a phase III trial compared to a phase II trial. In a phase III setting, where the aim is to provide confirmatory evidence for a new intervention, ensuring control of the type I error rate and reducing the chance and impact of bias will be high priorities. Although these are still important concerns in phase II settings, investigators may be more willing than regulators to apply/accept methods that risk inflation of error rate or statistical bias. Thus, the efficiency provided in a phase III platform trial may be more from operational efficiency compared to phase II where gains in both operational and statistical efficiency are possible.

Similarly, there might be differences in platform trials that are sponsored by a public sector institution and ones sponsored by industry. Regulatory issues will be more present in the latter—we refer the reader to the FDA draft guidance on master protocols for some further illustration of regulatory viewpoints [95]. Trials led by academic or public sector institutions will still need to follow this guidance if they are testing drugs: some concerns may be lessened, however, if trial results are not to be used for drug registration purposes. Several regulatory agencies provide design consultation advice, and this would be a useful route to take for researchers proposing a platform trial for registration purposes.

We have concentrated on frequentist concepts such as bias and type I error rate. Bayesian methods [96–98] are increasingly being utilized in the design and/or analysis of clinical trials; if a purely Bayesian analysis is being performed, then some statistical concerns may be lessened. However, even in a Bayesian trial that considers both the Bayesian design (e.g. Bayesian group sequential or multi-arm multi-stage designs [99–104], Bayesian sample size calculation [105–108], and adaptive randomization [109–113]) and analysis approaches [114], it is common to consider the chance of incorrectly recommending an ineffective treatment and to be interested in the estimated treatment effect from trial data alone. In this case, many of the statistical issues we discuss are still applicable. Further consideration of Bayesian versus frequentist approaches for specific statistical aspects in the context of adding arm is an interesting area for future work.

We have focused primarily on the statistical aspects of adding arms in this work. The optimal timing of adding and dropping arms in platform trials depends on the clinical context, the nature of the interventions, and the capability of stakeholders in delivering the amendments. It could be that a new arm is only added when an existing intervention arm is dropped, or the decision is independent of other adaptations. Adding and dropping arms too quickly may increase implementation complexity (and also increase the risk of type I or II errors) whereas acting slowly may reduce the benefits of these adaptive features. Practical guidance on deciding the timing of adding and dropping arms would help increase the uptake of the platform trial approach.

In conclusion, platform trials that allow adding of new arms provide great opportunities for improving the efficiency of evaluating interventions. Although several statistical issues are present, there are a range of methods available that allow robust and efficient design and analysis of these trials. Future research will undoubtedly add more and better methods to maximize the benefits provided by platform trials.

## Data Availability

Not applicable.

## Abbreviations

FWER: Family-wise error rate
FDR: False discovery rate
